# FDM Printability of PLA Based-Materials: The Key Role of the Rheological Behavior

**DOI:** 10.3390/polym14091754

**Published:** 2022-04-26

**Authors:** Rossella Arrigo, Alberto Frache

**Affiliations:** Dipartimento di Scienza Applicata e Tecnologia, Politecnico di Torino, Viale Teresa Michel 5, 15121 Alessandria, Italy; alberto.frache@polito.it

**Keywords:** additive manufacturing, 3D printing, FDM, PLA, polymer rheology

## Abstract

Fused deposition modeling (FDM) is one of the most commonly used commercial technologies of materials extrusion-based additive manufacturing (AM), used for obtaining 3D-printed parts using thermoplastic polymers. Notwithstanding the great variety of applications for FDM-printed objects, the choice of materials suitable for processing using AM technology is still limited, likely due to the lack of rapid screening procedures allowing for an efficient selection of processable polymer-based formulations. In this work, the rheological behavior of several 3D-printable, commercially available poly(lactic acid)-based filaments was accurately characterized. In particular, each step of a typical FDM process was addressed, from the melt flowability through the printing nozzle, to the interlayer adhesion in the post-deposition stage, evaluating the ability of the considered materials to fulfill the criteria for successful 3D printing using FDM technology. Furthermore, the rheological features of the investigated materials were related to their composition and microstructure. Although an exhaustive and accurate evaluation of the 3D printability of thermoplastics must also consider their thermal behavior, the methodology proposed in this work aimed to offer a useful tool for designing thermoplastic-based formulations that are able to ensure an appropriate rheological performance in obtaining 3D-printed parts with the desired geometry and final properties.

## 1. Introduction

Additive manufacturing (AM, also referred to as “3D-printing”) is an innovative computer-aided technology, which has gained a steadily increasing interest from both the academic and industrial worlds in recent years, due to a large number of advantages when compared to the traditional manufacturing processes usually employed for the production of polymer-based parts and objects [1,2,3]. In particular, the layer-by-layer approach of AM processes offers the opportunity to produce parts with complex geometries, or consolidated assemblies that are not attainable using traditional production processes for thermoplastics, such as extrusion or injection molding, while also reducing the amount of required material and waste produced [4,5]. Furthermore, the use of AM processes allows for the production of printed objects that are fully customizable, and tailored to obtain specific properties in an efficient and cost-effective way, overcoming the typical disadvantages of conventional technologies, such as the design and the cost of manufacturing the molds for the production of individual parts [6].

As far as the production of 3D-printed thermoplastics is concerned, material extrusion-based technologies, employing either granules or filaments as feedstocks, are the most exploited AM processes. These methods include different techniques, such as fused granular fabrication (FGF) [7], employing thermoplastic pellets similar to traditional injection molding, 3D plotting [8], 3D fiber deposition [9], and fused deposition modeling (FDM) [10]. This last is the most commonly used and well-established commercial process of manufacturing 3D-printed thermoplastics, and involves the layer-by-layer deposition of a molten thermoplastic filament, which is fed, in the solid state, through two counter-rotating rollers in a heated nozzle, moving according to a predetermined scheme [11]. The main advantages of FDM are the rapid and cost-effective production of customized components, the improved sustainability compared to traditional processing technologies, and the possibility of also using commodity materials for advanced applications, such as bio-medical devices, dental pieces, architectural structures, and automotive and aerospace parts, among others [12,13,14]. In addition, during the COVID-19 pandemic, FDM was exploited as an alternative method to produce personal protective equipment, including facial masks and shields, further demonstrating the great potential of this AM technology [15].

**Figure 1:**
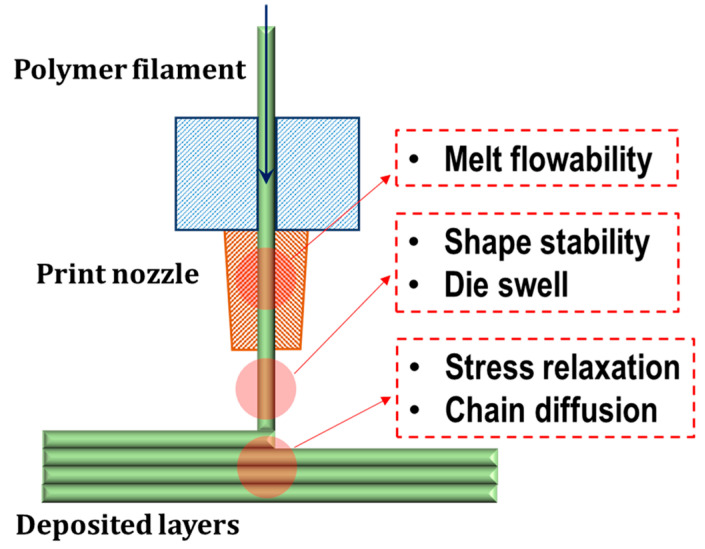
Schematic of FDM process and rheological characteristics of melt filament affecting the different processing stages.

Despite the great versatility of the FDM process, the catalog of available materials is still severely limited, mainly due to the lack of reliable information on the processability of thermoplastics in this forming technology [16]. Therefore, in order to widen the portfolio of thermoplastic-based formulations suitable for 3D-printing applications, there is a need for easy and straightforward methods that allow for an efficient optimization of the material composition and characteristics, overcoming the disadvantages related to the use of ineffective and time-consuming trial and error procedures [17]. In this context, given that the material processability is strictly governed by its rheological behavior, a screening process for the assessment of the FDM processability of polymeric materials has to take into account the polymer rheological features, and the related critical implications, during all steps of a typical FDM process [18,19]. Looking at the scheme reported in Figure 1, different requirements need to be satisfied in order to classify a material as “FDM-printable”. First, the polymer filament must be able to be extruded through the printing nozzle, showing viscosity values low enough to ensure the flowability of the melt [20]. Second, during the deposition step when the melt exits the nozzle, the extruded material must retain its shape under gravity, and under the stresses generated by the deposition of subsequent layers; therefore, a marked shear thinning behavior, involving a sudden increase in the melt viscosity with a decrease in the shear rate, is required in order to avoid shape instability and defect formation [21]. Finally, in the post-deposition stage, polymer chains, lying at the interface between adjacent deposited layers, must be able to interdiffuse across the interface, to ensure a good level of interlayer adhesion and, hence, adequate mechanical performances by the printed part [22].

In this work, several commercially available poly(lactic acid) (PLA)-based filaments for FDM, containing different types of additives and/or solid fillers, were thoroughly characterized from a rheological point of view, aiming to develop an easy and affordable procedure to model and predict the FDM processability of thermoplastic materials. In particular, an accurate analysis of the rheological behavior of the materials was carried out, considering the different steps of a typical FDM process, illustrated above, in order to establish useful relationships between the material properties and FDM printability. Lastly, selected formulations were used to produce printed parts, and the obtained microstructures were discussed and related to the processability of the different materials.

## 2. Materials and Methods

### 2.1. Materials

The following commercially available PLA-based filaments were used:

- Ecogenius PLA (TreeD Filaments), main characteristic: tensile modulus 65.5 MPa;
- Wound Up—coffee-filled filament (3D-FUEL), which is a composite material based on PLA, and filled with by-products from the coffee roasting process. Main characteristics: maximum tensile strength 34 MPa, tensile elongation 4.1%;
- Buzzed—beer-filled filament (3D-FUEL), which is a composite material based on PLA and by-products from the beer malting process. Main characteristics: maximum tensile strength 36 MPa, tensile elongation 5.8%;
- Shogun heat resistant PLA (Treed Filaments), main characteristics: yield tensile strength 38 MPa, tensile elongation > 10%;
- Kyotoflex BioFlex (Treed Filaments), main characteristics: tensile strength 19.6 MPa, maximum elongation 450%;
- Entwined—hemp-filled filament (3D-FUEL), which is a PLA-based composite containing short hemp fibers. Main characteristics: maximum tensile strength 38 MPa, tensile elongation 4.6%.

In Table 1, all materials used, the type of additives contained in the formulation (when declared by the supplier), and the code used hereinafter are listed. All the filaments have a diameter of 1.75 mm.

### 2.2. Methods

The rheological behavior of all investigated materials was investigated using an ARES (TA Instrument, New Castle, DE, USA) strain-controlled rheometer, equipped with a parallel plate geometry (plate diameter = 25 mm, typical gap imposed during the tests = 1 mm). The following tests were carried out:

- Strain sweep measurements at 200 °C and frequency (ω) = 1 rad/s;
- Frequency sweep measurements at 200 °C, ω in the range 0.1–100 rad/s. The value of strain amplitude was fixed low enough to ensure the recorded response was within the linear viscoelastic region of investigated materials. Data collected through frequency sweep tests were fitted using the following models. In particular, the Carreau model (Equation (1)) and a modified Carreau model (Equation (2)) were used to fit data for materials showing Newtonian and non-Newtonian rheological behavior, shown respectively as:
  (1) $$\eta \;\left(\omega \right)=\eta_{0}{\left[1+{\left(\lambda \omega \right)}^{2}\right]}^{\frac{n-1}{2}}$$
  (2) $$\eta \;\left(\omega \right)=\eta_{0}{\left[1+{\left(\lambda \omega \right)}^{2}\right]}^{\frac{n-1}{2}}+\frac{\sigma_{0}}{\omega }$$
  where $\eta_{0}$ is the zero-shear viscosity, λ is the relaxation time, *n* is a parameter related to the slope of the viscosity curve in the shear thinning region, and $\sigma_{0}$ is the yield stress.
- Linear stress relaxation tests at 200 °C, submitting the sample to a single-step strain at γ_0_ = 1%.

All the measurements were performed under inert N_2_ atmosphere, to avoid material oxidative degradation during the tests. Prior to the measurements, the samples were vacuum dried at 60 °C for 4 h.

Specimens for the rheological characterizations were obtained from pelletized filaments through a compression molding step, using a laboratory press (Teach Line 200 T Collin, Ebersberg, Germany) working at 190 °C with an applied pressure of 100 bar for 2 min. Prior to the processing, the materials were vacuum dried at 60 °C for 4 h.

Selected filaments were 3D printed using an open chamber FDM machine (One 3D printer Roboze, Bari, Italy), with a printing plate of 28 cm × 34 cm equipped with a 0.4 mm nozzle. During the processing, the filament spools were maintained at 40 °C in a drying chamber. The following processing conditions were adopted: nozzle temperature = 200 °C, bed temperature = 65 °C, printing speed = 30 mm/s, filling = 50%, layer thickness = 0.2 mm, and raster angle = +45°/−45°.

The morphological characterization of the investigated materials was performed using a Zeiss (Oberkochen, Germany) VP scanning electron microscope (beam voltage: 20 kV). Before observation, the materials were cryogenically fractured in liquid nitrogen and the obtained surfaces were gold metallized.

## 3. Results and Discussion

As briefly discussed previously, the knowledge and the monitoring of the rheological behavior of the material in each step of the process is of crucial importance, in order to gain fundamental insights into the printability of thermoplastic polymers. In the sections below, the rheological characteristics of the investigated materials, associated with the different steps of a FDM process, are discussed, specifically considering the following main aspects: the flow of the melt in the nozzle and its extrusion, the maintenance of the shape, the integrity of the extrudate in the stand-off region and during the layer-by-layer deposition, and the welding of the deposited layers through polymer chain interdiffusion.

### 3.1. Flow Behavior in the Nozzle

**Figure 2:**
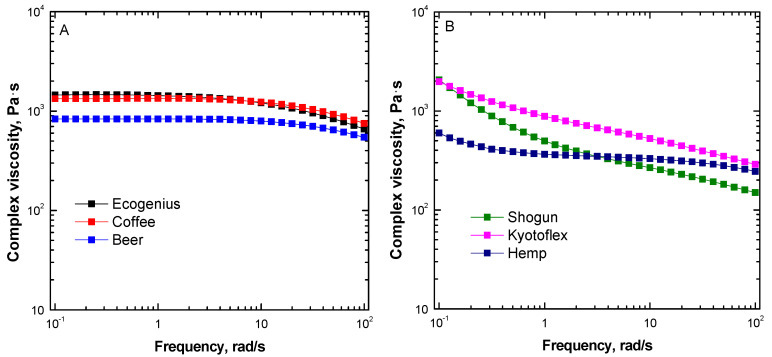
Complex viscosity curves for PLA-based materials showing (**A**) Newtonian plateau, and (**B**) yield stress.

In order to assess the flowability of the melts in the printing nozzle, and their behavior during the extrusion step, the complex viscosity of the investigated materials was evaluated using dynamic frequency sweep tests, and the obtained results are plotted in Figure 2A,B. Looking at the obtained trends of the complex viscosity as a function of frequency, it is clear that the studied materials exhibit very different rheological behavior.

In particular, the Ecogenius, coffee, and beer samples, whose complex viscosity curves are reported in Figure 2A, show marked Newtonian behavior at low and intermediate frequencies, with a Newtonian plateau developing for most of the investigated frequency range, followed by a mild shear thinning at high frequencies. Conversely, the Shogun, Kyotoflex, and hemp samples (curves depicted in Figure 2B) exhibit non-Newtonian behavior in the low frequency region, involving a sudden increase in the complex viscosity as the frequency decreases, and a more pronounced shear thinning compared to the samples shown in Figure 2A. The differences observed in the rheological behavior of the investigated materials is related to their microstructure and, more specifically, to the influence of the different additives and/or fillers contained in the commercial formulations on the dynamics of the PLA macromolecules. In particular, the materials showing Newtonian plateau contain no additives (Ecogenius), or low molecular weight species (coffee and beer), which are not able to modify the relaxation dynamics of PLA chains; therefore, their rheological behavior is nearly unchanged with respect to what is typically expected for an unfilled PLA sample, which usually shows a fully developed Newtonian plateau at low and intermediate frequencies [23]. On the other hand, the disappearance of the Newtonian plateau for the Shogun, Kyotoflex, and hemp samples is associated with the presence of embedded solid fillers, possibly interacting strongly with the matrix, which interfere with the relaxation processes of the polymer macromolecules, hampering their complete relaxation. This phenomenon causes the appearance of an apparent yield stress behavior at low frequencies, and the amplification of the shear thinning characteristics in the high frequency region [24]. In addition, some differences emerge when comparing the rheological behavior of these filaments. In particular, the trend of the complex viscosity curve of the hemp sample is very similar to those of the samples showing a Newtonian plateau, as this material also shows a plateau at intermediate frequencies (with an increase in the complex viscosity only at the lowest tested frequencies) and a moderate shear thinning. This result is explained considering the fact that the hemp filament contains low aspect ratio organic fillers (i.e., short hemp fibers), which are not able to significantly alter the dynamics of PLA chains. As a matter of fact, the rheological behavior of the material is governed by that of the matrix, while the effect of the embedded fillers is only appreciable at very low frequency values. For the Shogun and Kyotoflex samples, the introduction of high contents (namely, >15 wt.%) of anisotropic solid fillers promotes the appearance of remarkable non-Newtonian behavior, and the complex viscosity of these materials continuously decreases over the investigated frequency range.

It was demonstrated that a pronounced shear thinning behavior is beneficial for the processability of polymers in extrusion printing processes [25,26]. In fact, a prominent shear thinning ensures low viscosity values within the nozzle, where the polymer is subjected to high shear rates, facilitating the extrusion step and reducing the pressure required to extrude the material. On the other hand, marked shear thinning characteristics guarantee a rapid increase in the material viscosity at the nozzle exit, where the shear rate drops exponentially, thus avoiding material oozing, and facilitating the retainment of the extrudate shape at the beginning of the stand-off region. This phenomenon is particularly evident when the material rheological behavior involves the appearance of an evident yield stress at low frequencies, further promoting the shape stability of the extrudate, which is discussed in detail in the following section.

**Figure 3:**
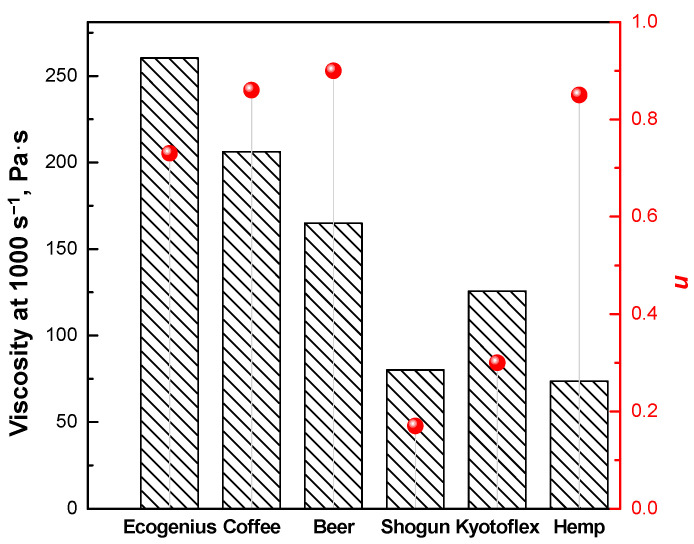
Viscosity values (bars) calculated at the actual shear rate value (i.e., 1000 s^−1^) experienced during the flow in the printing nozzle, and values of the parameter n of the Carreau model (red symbols) for all investigated PLA-based materials.

To quantify the magnitude of the shear thinning behavior, and to discriminate between the differences observed for the investigated PLA-based filaments, the determination of the slope of the viscosity curve in the shear thinning region is useful. To this aim, the viscosity data plotted in Figure 1 were fitted with either a Carreau (Equation (1)), or Carreau modified model (Equation (2)), depending on the presence of a Newtonian plateau or yield stress behavior, respectively, and the obtained fitting parameters are listed in Table 2. Looking at the values of the parameter *n,* represented also in Figure 3, it is evident that materials showing a Newtonian plateau (i.e., Ecogenius, coffee, and beer), as well as the hemp sample, exhibit *n* values approaching 1, which indicate a mild decrease in the viscosity as a function of shear rate. Conversely, the *n* values of the Kyotoflex and Shogun samples are significantly lower, confirming the more significant shear thinning behavior already noticed for these materials.

It is important to know the actual values of viscosity of the samples during a typical FDM process, which allows for the assessing of the processability of the considered materials during the extrusion step. To do this, the value of the actual shear rate undergone by the materials when they pass through the nozzle was calculated, according to a procedure reported elsewhere [27]. Considering a filament diameter of 1.75 mm, a nozzle diameter of 0.4 mm, and a printing speed of 30 mm/s, a value for the shear rate of about 10^3^ s^−1^ was found. Using Carreau and modified Carreau models (Equations (1) and (2)), the viscosities at such values of the shear rate were derived, and the obtained results are reported in Figure 3.

As expected, the viscosity of the filaments showing a Newtonian plateau and mild shear thinning is significantly higher compared to that of the materials exhibiting pronounced shear thinning. This feature indicates significant differences in the processability of the different filaments in the extrusion step; in particular, for the materials showing high viscosity at the typical shear rates involved in the flow through the nozzle, more difficult extrusion is predicted, compared to the samples with prominent shear thinning, and higher pressure values are required to efficiently extrude the filaments.

**Figure 4:**
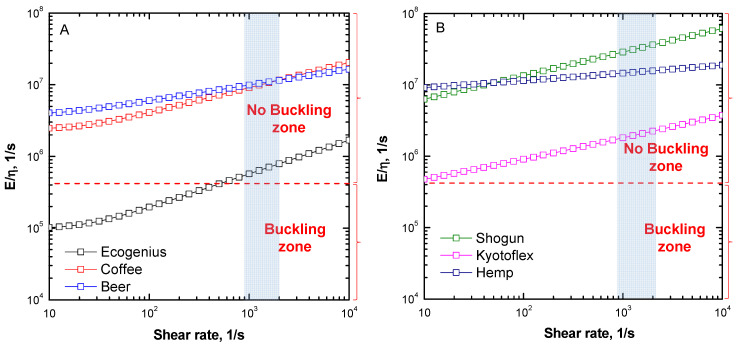
Typical buckling analysis performed on PLA-based materials showing (**A**) Newtonian plateau, and (**B**) yield stress.

A further issue usually encountered during the extrusion step is the phenomenon of filament buckling, which occurs when the material is not stiff enough to resist the pressure applied during the feeding of the filament through the nozzle. Since the required pressure is proportional to the melt viscosity, the concerns related to buckling are particularly important for materials showing high viscosities during the flow in the nozzle. More specifically, filament buckling occurs when the applied pressure exceeds a critical stress, which is related to the mechanical properties of the solid filament and its *slanderness* ratio, i.e., the ratio between its radius and the length of the filament withstanding the roller pressure [28]. Venkataraman et al. [29] developed a method for the prediction of the filament buckling, by calculating the critical buckling stress using Euler’s analysis for the pin-ended boundary condition, and comparing the obtained values to the pressure required to extrude the filament. In particular, the proposed method predicts buckling if the ratio of the elastic modulus (E) and the viscosity of the material falls below a certain critical value, which is typically in the range of 3 × 10^5^ to 5 × 10^5^ s^−1^ [30]. Figure 4A,B reports the values of the calculated E/η ratios for all investigated PLA-based filaments as a function of shear rate. In the Figures, the dotted lines represent the critical stress value calculated according to the method proposed by Venkataram [29]. In the region in which the E/η is higher than the critical stress, the material should not present buckling issues; otherwise, filaments falling in the lower region are likely to fail during extrusion, due to buckling. In Figure 4, the range of shear strain involved in a FDM process (under the conditions already stated, i.e., filament diameter = 1.75 mm, nozzle diameter = 0.4 mm, and printing speed = 30 mm/s) is also highlighted. For the PLA-based filaments in this study, the adopted calculation method predicts no buckling at the selected processing conditions. Nevertheless, looking at the curves reported in Figure 4, some differences emerge. In particular, the curve of the Ecogenius sample lies in proximity to the critical buckling stress zone in the considered shear rate interval, due to the high viscosity of this material during the extrusion step; however, the curves of the coffee and beer samples are entirely in the no-buckling region for the whole tested shear rate range. This feature is related to the low viscosity of these samples, compared to the Ecogenius sample, but also to their high rigidity. As far as the behavior of the non-Newtonian filaments is considered (Figure 4B), the curves of all materials fall in the no-buckling zone for all investigated shear rates, due to both low viscosity during the extrusion step, and high elastic modulus values guaranteed by the reinforcing action provided by the embedded solid fillers. 

### 3.2. Shape Stability and Die Swell

**Figure 5:**
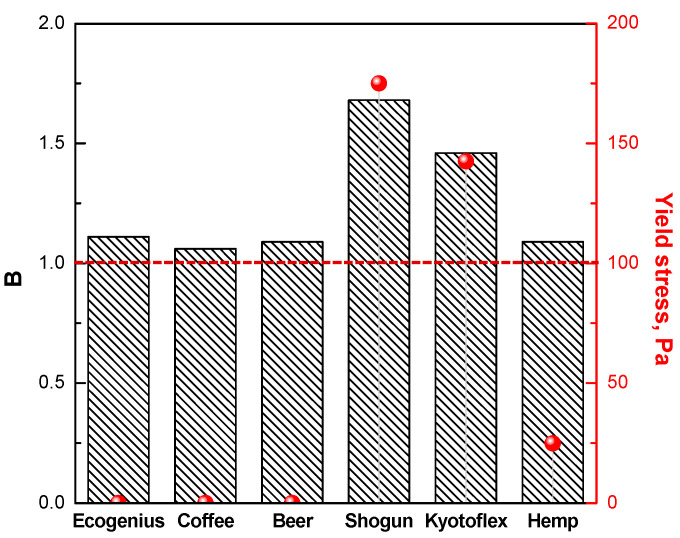
Calculated die swell (bars) and yield stress values (red symbols) for all investigated PLA-based filaments.

As briefly anticipated previously, when the melt filament exits the printing nozzle, it is of fundamental importance that the material shows low propensity to drip; furthermore, to obtain a good quality of the printed part, the filament should maintain its shape under gravity for the whole stand-off region, and under the stress generated by the layers subsequently deposited during the deposition step. Both these requirements are ensured if the material exhibits yield stress in quasi zero-shear conditions, i.e., at low frequencies/shear rates. Recalling the viscosity curves depicted in Figure 2B, the Shogun, Kyotoflex, and hemp samples exhibit an appreciable yield stress in the low frequency region (the values of yield stress were calculated through the fitting of the data with a modified Carreau model and are reported in Figure 5), allowing a sudden increase in the viscosity when the shear rate tends to 0. This behavior is associable to a melt structuring action of the solid fillers present in these filaments, and guarantees a good shape stability of the extrudate at the exit of the nozzle, when the shear rate exponentially decreases, and also during and after the deposition step. Furthermore, the occurrence of yield stress is required to avoid the premature extrusion and the dripping of the material prior to the beginning of the processing, during the step of pre-heating of the nozzle, when the filament is already housed within the nozzle. Conversely, the Ecogenius, coffee, and beer samples exhibit a fully developed plateau in the low frequency region, with no yield stress; for these materials, the viscosity does not increase when the shear rate tends to zero, and this issue could compromise the shape stability of the extrudate, thus affecting the quality and the surface topography of the printed part after processing.

Similar to other polymer processing technologies, in which the melt is forced to pass through an orifice with a small diameter, the extrudate swelling (or die swell) at the exit of the nozzle also needs to be taken into account in the FDM technique. This phenomenon is caused by the normal stresses developed perpendicularly to the extrusion direction (due to the viscoelastic feature of the polymer melts), which relax when the macromolecular chains return to their original state once extruded through the nozzle. The relaxation of the stored elastic energy leads to a radial expansion of the melt, which results in the obtainment of an extrudate with a diameter greater than that of the nozzle. Extrudate swelling is usually quantified through the parameter B, representing the ratio between the actual diameter of the extrudate and that of the nozzle. The die swell phenomenon is remarkable for FDM processing, as high B values negatively affect the surface resolution of the printed part.

The die swell is difficult to measure, due to the progressive thinning of the melting filament exiting the extrusion orifice; therefore, different methods were developed to predict this phenomenon, using rheological data collected through dynamic frequency sweeps measurements. In particular, Tanner et al. [31] demonstrate the possibility of calculating the die swell considering the zero-shear viscosity, the relaxation time of the melt, and the wall shear stress experienced by the polymer during the extrusion through the nozzle. Using the zero-shear viscosities, and relaxation times derived from the fitting of the viscosity data (values reported in Table 2), the theoretical values of B were calculated for all investigated filaments, and the obtained results are presented in Figure 5. It is interesting to highlight that, given that the extrudate swell phenomenon is related to the elastic component of the viscoelastic behavior of polymer melts, and to the relaxation processes of the melt, lower values of B are expected for materials showing a Newtonian plateau, for which the relaxation of the macromolecules is fully completed. In fact, the Ecogenius, coffee, beer, and hemp samples exhibit B values close to 1, indicating an almost irrelevant increase in the extrudate diameter with respect to that of the nozzle. However, the B values calculated for the Shogun and Kyotoflex samples are 1.65 and 1.42, respectively, suggesting that for a successful processability of these samples, an accurate optimization of the relevant processing conditions and variables (such as nozzle temperature, feeding rate, and length of the nozzle) must be performed, in order to minimize extrudate distortion.

### 3.3. Interlayer Adhesion and Welding

**Figure 6:**
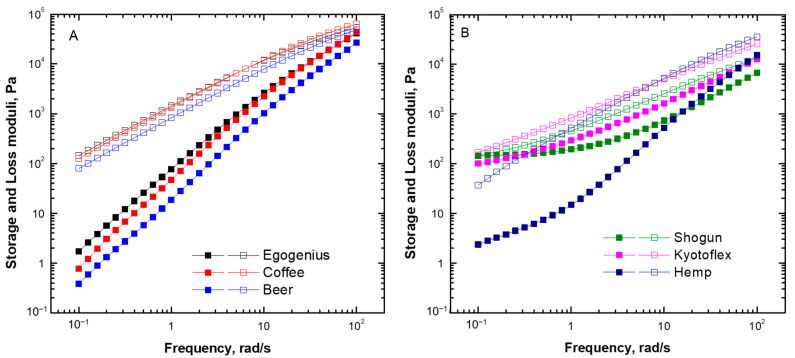
Dynamic moduli as a function of frequency for PLA-based materials showing (**A**) terminal, and (**B**) non-terminal rheological behavior (G′ full symbols, G′′ empty symbols).

During the last step of FDM processing, the extruded filaments are deposited on the printed bed, or on the already deposited layers, and solidify. This stage plays a key role in the determination of the final mechanical properties of the printed part, as they depend on the interlayer adhesion between the layers deposited subsequently. In this context, the diffusion of the polymer chains across the interface and their relaxation (i.e., the re-arrangement of the macromolecules towards their original state) dictate the interlayer bonding and the welding [32]. It is demonstrated that the macromolecular interdiffusion leading to successful welding is controlled by the prominence of the elastic or viscous component of the viscoelastic behavior of the melt, and on the relaxation time of the polymer [33]. In particular, the chain interdiffusion at the interface between the layers is favored in materials showing a predominantly viscous behavior at the welding temperature [34]. In other words, an improved interlayer adhesion is expected if the viscous modulus (G′′) of the melt has higher values that the elastic one (G′) in the terminal region. In Figure 6A,B, both dynamic moduli of all investigated filaments measured using frequency sweep tests are reported. Although all the filaments fulfil the requirement G′′ > G′, the viscoelastic behavior of the Shogun and Kyotoflex samples is significantly different compared to that of the other studied systems. In fact, for both filaments, the curve of G′ tends to become frequency independent, due to the arrestment of the macromolecular dynamics caused by the presence of embedded solid fillers. This feature leads to an approaching of the curves of the two dynamic moduli, and G′ and G′′ present very close values in the terminal region.

Nevertheless, it was shown that a more accurate procedure to predict interlayer bonding and welding concerns the application of the so-called Dahlquist criterion, originally developed for the study of the welding performance of adhesives and successfully applied to 3D printing processes [35,36]. This criterion establishes that there is a critical value of the elastic modulus (equal to 3 × 10^5^ Pa), below which the material shows instantaneous adhesion, irrespective of the prominence of the elastic or viscous modulus. Obviously, it is important to consider the temperature at which the adhesion is evaluated, which depends not only on the printing conditions but also on the thermal conductivity of the polymer. Usually, considering the typical thermal conductivity values of polymeric materials, the cooling time needed to reach the conditions dictated by the Dahlquist criterion is less than 1 s [35].

**Figure 7:**
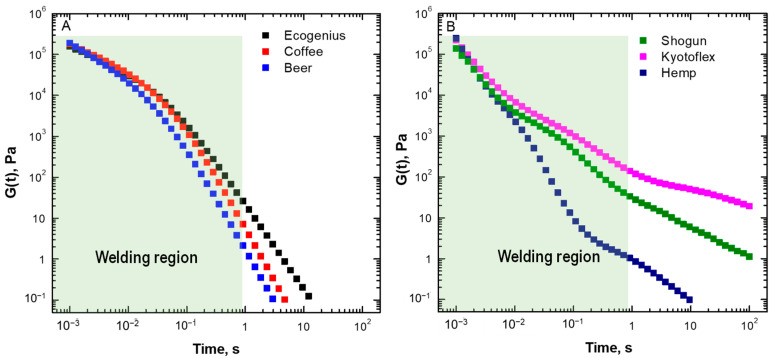
Stress relaxation modulus as a function of time for PLA-based materials showing (**A**) complete, and (**B**) incomplete relaxation. The green area highlights the region in which (according to the Dahlquist criterion) interlayer bonding is expected.

To verify the interlayer adhesion of the investigated PLA-based filaments, stress relaxation tests were then performed, and the obtained results are plotted in Figure 7A,B. The green region highlighted in the figure represents the so-called welding zone, according to the aforementioned criterion, taking as reference a modulus of 3 × 10^5^ Pa and a cooling time of 1 s. Despite the differences between the behavior of the samples reported in Figure 7A,B, attributable to the complete relaxation of the materials showing more pronounced Newtonian behavior, and the retarded chain dynamics resulting from the presence of the fillers in the Shogun, Kyotoflex, and hemp samples, all considered filaments fulfil the Dahlquist criterion, as the relaxation moduli curves of all materials lie in the welding region at the considered printing temperature (i.e., 200 °C). For all tested materials, therefore, an acceptable degree of interlayer adhesion is expected, leading to a good extent of layer bonding and welding. However, an evaluation of the welding between the subsequently deposited layers based solely on rheological features is not exhaustive, as the mechanism underlying the layers adhesion strongly depends on the kinetics of cooling, solidification, and possible crystallization of the material [37,38]. As an example, Nogales et al. [39] evaluate the evolution of the microstructure in an isotactic polypropylene sample during a FDM process, and reveal a variation of the crystallinity across the individual layers. More specifically, the results document that the polymer is more crystalline in the bulk of the layer and less crystalline in the vicinity of the interfaces, and this feature further facilitates the welding between the layers favoring the chain interdiffusion at the interface. In the case of PLA-based materials, due to the intrinsically low crystallinity of the matrix, it is expected that the deposited layers exhibit a highly amorphous microstructure. Therefore, for the filaments studied in this work, it is inferred that the adhesion between the layers is mostly affected by the rheological behavior of the melt (in terms of the stress relaxation dynamics of PLA macromolecules and the prominence of the viscous component), while the crystallization kinetic and mechanism are less decisive factors.

### 3.4. Printability

To sum up, the rheological properties of the selected PLA-based filaments relevant to the different stages of a typical FDM process were evaluated, and in Table 3 the main results are summarized, considering a fail/pass procedure involving all the considered rheological characteristics.

Considering the results reported in Table 3, materials showing more pronounced non-Newtonian characteristics fulfill most of the rheological requirements for FDM processability, especially the Shogun and Kyotoflex samples, which exhibit the best performance in terms of flowability through the nozzle, and shape maintenance in the stand-off region and during the deposition stage. Although these materials show the higher values of die swell, this feature is less significant compared to the other considered properties, as the effects due to the swelling of the extrudate at the exit of the nozzle are easily minimized by properly adjusting the process variables. At variance, the requirements related to the flowability and to the shape stability are the most significant, as the viscosity, shear thinning behavior, and yield stress are mostly related to the material microstructure, making the proper modulation of these characteristics through the optimization of the processing conditions more difficult. 

**Figure 8:**
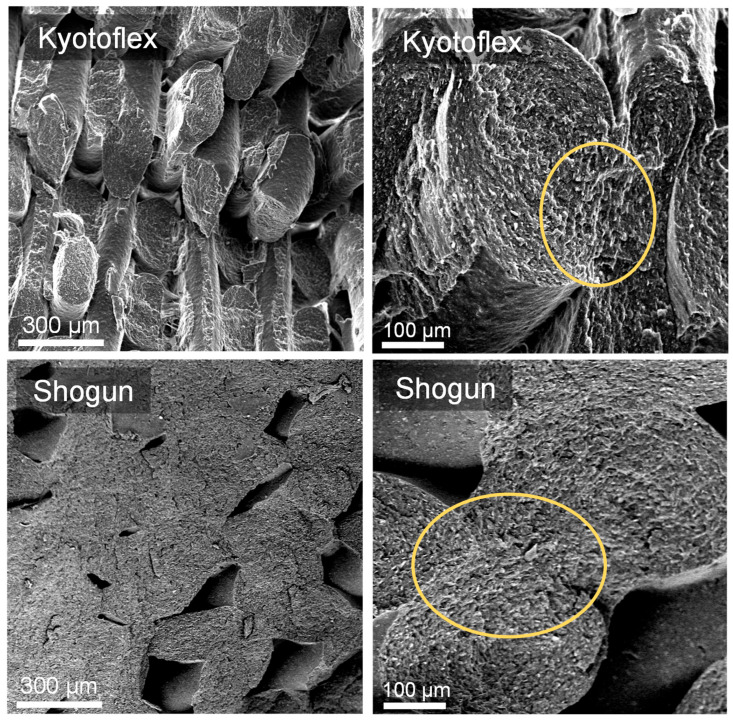
Representative SEM micrographs at different magnifications of selected 3D-printed parts.

Aiming at verifying the effectiveness of the proposed procedure, based on the analysis of the filament rheological behavior, the Shogun and Kyotoflex samples were selected to be processed through FDM, and the morphology of the printed parts was investigated through SEM observations. Representative micrographs collected at different magnifications are showed in Figure 8. For the printed Kyotoflex samples, the typical microstructure of the layer-by-layer deposition is still visible, notwithstanding the good extent of interlayer adhesion. In the case of the Shogun printed parts, the single deposited layers are hardly distinguishable, and a more compact microstructure, with a lesser extent of voids, was achieved. Although similar rheological behavior is shown by the two materials, the differences observed in the microstructure seem to be related to a different degree of interlayer bonding between adjacent layers. To gain further insight into the 3D printability of the investigated samples, their thermal behavior should be considered, as the phenomena underlying the FDM processability of polymer filaments are also strongly dependent on the behavior of the material during the cooling from the printing temperature to room temperature, as well as on the crystallinity content at the end of the process.

## 4. Conclusions

In this work, the rheological behavior of several PLA-based filaments suitable for FDM processing was thoroughly investigated, aiming at developing an easy procedure to predict the FDM processability of thermoplastic materials. In particular, different rheological characteristics, relevant for the different steps of a typical FDM process, were measured or calculated using proper models, and the obtained results were discussed considering the microstructure of the investigated samples.

As far as the flowability of the materials through the printing nozzle is considered, materials showing more pronounced non-Newtonian characteristics (involving a remarkable shear thinning at high shear rates) exhibit an improved extrudability, since a prominent shear thinning behavior ensures low viscosity values during the flow through the nozzle at high shear rates, and a rapid increase in the viscosity at zero-shear conditions, i.e., when the material exits the nozzle. In particular, the presence of a yield stress behavior is preferred, as it guarantees the absence of the dripping phenomena at the exit of the nozzle, and shape stability of the filament in the stand-off region and during the deposition stage. Furthermore, high viscosity values at the typical shear rates of the extrusion through the nozzle could result in filament buckling issues, causing distortion of the filament, and a failure of the extrusion stage. However, marked non-Newtonian features promote significant extrudate swelling of the filaments exiting the nozzle; therefore, an accurate optimization of the processing conditions is required to avoid shape distortions. Finally, to ensure a good extent of interlayer adhesion and bonding, a prominent viscous behavior is preferable, as a marked elastic component or an incomplete relaxation of the polymer chains during the cooling of the material after the deposition affects the welding between the subsequently deposited layers, negatively influencing the mechanical properties of the printed part.

## Figures and Tables

**Table 1:** Commercial names, additives, and codes of all investigated PLA-based filaments.

Material	Additives	Code
PLA Ecogenius	None	Ecogenius
Wound Up coffee-filled PLA	Byproducts from coffee roasting	Coffee
Buzzed beer-filled PLA	Byproducts from beer malting	Beer
Shogun heat resistant PLA	Mineral filler (likely talc) ^1^	Shogun
Kyotoflex Bioflex PLA	Elastomer in blend and mineral fillers (likely talc and calcium carbonate) ^1^	Kyotoflex
Entwined—hemp filled	Short hemp fibers	Hemp

^1^ Information not available from the supplier and obtained through preliminary analyses as specified in Supplementary Information, Table S1, and Figures S1 and S2.

**Table 2:** Fitting parameters of complex viscosity curves using Carreau (Equation (1)) or Carreau modified (Equation (2)) models.

Sample	$\eta_{0}\;[\mathrm{Pa}\cdot s]$	λ [s]	*n*	$\sigma_{0}\;[\mathrm{Pa}]$
Ecogenius	1499	0.010	0.73	--
Coffee	1365	0.008	0.86	--
Beer	843	0.005	0.90	--
Shogun	335	0.200	0.17	175.0
Kyotoflex	599	0.110	0.30	142.5
Hemp	338	0.005	0.85	24.9

**Table 3:** Results of fail/pass procedure concerning the rheological characteristics of investigated filaments.

Sample	Flowability	Buckling	Shape Stability	Die Swell	Interlayer Adhesion
Ecogenius	NO	≈	NO	OK	OK
Coffee	NO	OK	NO	OK	OK
Beer	NO	OK	NO	OK	OK
Shogun	OK	OK	OK	≈	OK
Kyotoflex	OK	OK	OK	≈	OK
Hemp	OK	OK	≈	OK	OK

## Data Availability

The data presented in this study are available on request from the corresponding author.

## Supplementary Materials

The following supporting information can be downloaded at: https://www.mdpi.com/article/10.3390/polym14091754/s1, Table S1: Main results from DSC and TG analyses for PLA-based filaments; Figure S1: ATR-FTIR spectrum and SEM micrograph of Kyotoflex filament; Figure S2: ATR-FTIR spectrum and SEM micrograph of Shogun filament.
