# Supplementary material for: FDM Printability of PLA Based-Materials: The Key Role of the Rheological Behavior
Source: Polymers (Basel). 2022 Apr 26;14(9):1754. doi: 10.3390/polym14091754 (PMC9104839; doi:10.3390/polym14091754)
Supplement: Supplementary file 1 [file polymers-14-01754-s001.zip › polymers-1699006-supplementary.pdf]

Supplementary Materials:
FDM printability of PLA based-materials: the key role of the rheological behavior

Rossella Arrigo*, Alberto Frache

The investigated filaments were characterized through DSC analyses, aiming at ssessing the possible effect of the different additives on the PLA thermal behavior. The measured characteristics temperatures (i.e glass transition -Tg - and melting -Tm- temperatures) are listed in Table S1.
Furthermore, studied materials were subjected to thermogravimetric analyses and the onset temperature for thermal degradation phenomena and the residues remaining at the end of the tests are reported in Table S1.

Table S1. Main results from DSC and TG analyses for PLA-based filaments.

**Sample**	**T_g [°C]**	**T_m [°C]**	**T_{onset} [°C]**	**Residue [%]**
Ecogenius	56.6	152.1	315.4	< 2
Coffee	59.9	151.4	323.4	< 2
Beer	59.3	150.1	295.5	< 2
Shogun	55.0	114.6	326.2	17.0
Kyotoflex	-31.2; 57.2	155.0	339.5	28.3
Hemp	58.7	150.8	296.5	< 2

As clearly observable in Table S1, in Shogun and Kyotoflex samples a higher residue at the end of TGA was found, as compared to other filaments. Furthermore, the results of the DSC chaacterization of Kyotoflex indicate the presence of a second polymer in blend with PLA, having a significantly lower glass transition temperature with respect to that of the matrix.
In order to investigate the nature of the additives and/or solid fillers contained in Kyotoflex and Shogun, these materials were subjected to SEM and ATR-FTIR analyses.
Figures S1, reports the results of the characterizations for Kyotoflex sample. The informations acquired through DSC, TGA, SEM and EDX characterizations suggest that this filaments contains an elastomer in blend with PLA, as well as embedded talc and calcium carbonate particles.
Results form ATR-FTIR and SEM characterizations performed on Shogun filament are reported in Figure S2. Performed caracterizations indicates that Shogun filaments contains about 17.0 wt.% of talc particles.

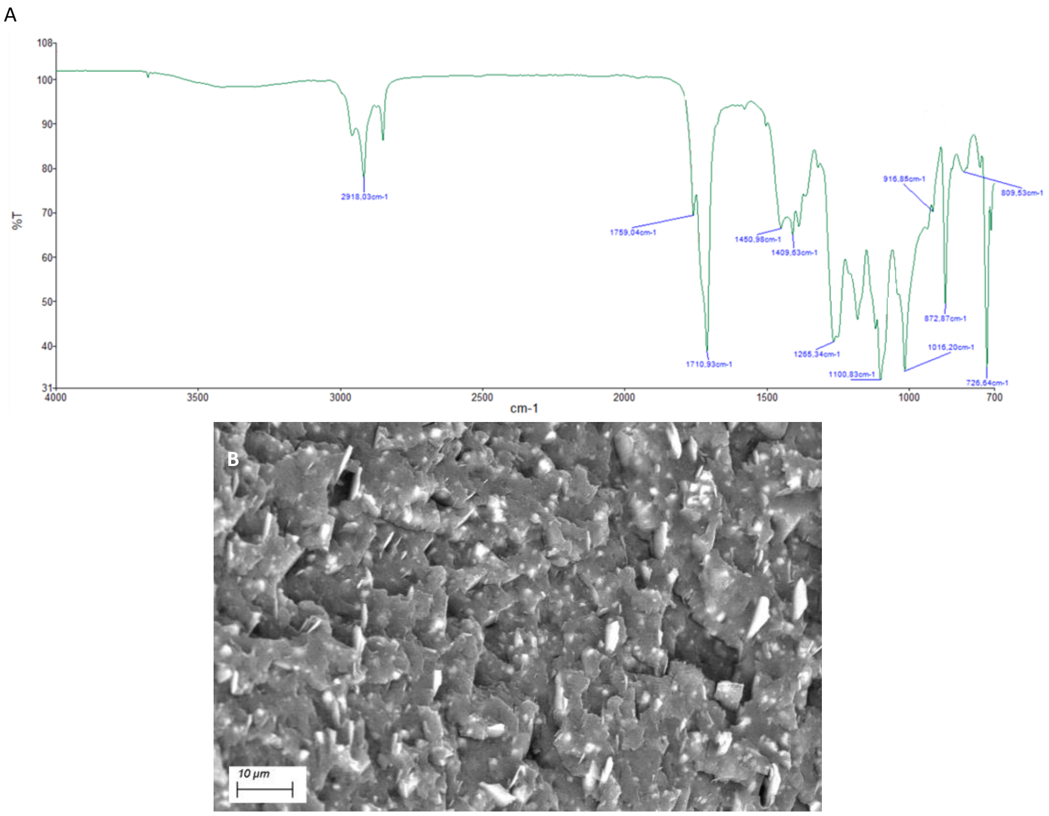

Figure S1. ATR-FTIR spectrum and SEM micrograph of Kyotoflex filament.

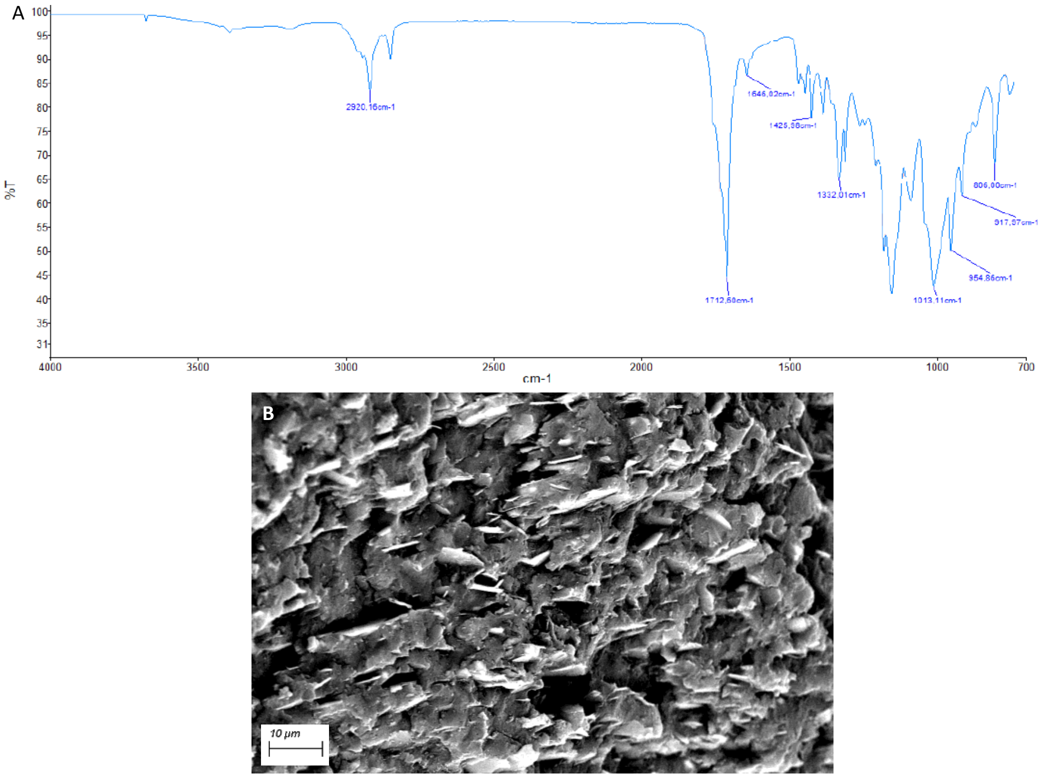

Figure S2. ATR-FTIR spectrum and SEM micrograph of Shogun filament.

Methods:

DSC analyses were performed on weighted samples of about 8 mg placed in sealed aluminum pans using a Q20 TA Instrument (New Castle, DE, USA). All the experiments were performed under dry N2 (20 ml/min). The samples were subjected to the following cycle: a heating ramp from − 50 to 200 °C, a cooling ramp from 200 to − 50 °C, and a second heating ramp from − 50 to 200 °C. All the heating/cooling ramps were performed at a scanning rate of 10 °C/min.

Thermogravimetric analyses (TGA) were performed using a Discovery apparatus (TA Instruments, USA) (experimental error: ±0.5 wt%, ±1 ◦C). Samples (about 10 mg) were placed in alumina pans and runs were carried out in the range 50–700 ◦C, with a heating rate of 10 ◦C/min, under nitrogen flow (35 mm/min).

ATR-FTIR spectra were collected by means of a Perkin Elmer Spectrum 100 spectrometer (Shelton, Connecticut, USA) equipped with an attenuated total reflection (ATR) diamond probe. FTIR spectra were recorded at wavelengths from 700 to 4000 cm^{-1}, with 4 cm^{-1}resolution; 16 scans were collected.

An EVO 15 scanning electron microscope (SEM) from Zeiss (Oberkochen, Germany), coupled with Ultim Max 40 energy dispersive X-ray (EDX) micro-analyzer by Oxford Instruments (High Wycombe, UK) was used for assessing the morphology of the filaments.
